# Student learning in interprofessional practice-based environments: what does theory say?

**DOI:** 10.1186/s12909-015-0492-1

**Published:** 2015-11-26

**Authors:** Chris Roberts, Koshila Kumar

**Affiliations:** Sydney Medical School, Sydney University, Sydney, Australia; Flinders University Rural Clinical School, Adelaide, Australia

**Keywords:** Interprofessional education, Practice-based learning, Patient care team, Professional identity

## Abstract

Student learning in interprofessional practice-based environments has garnered significant attention in the last decade, and is reflected in a corresponding increase in published literature on the topic. We review the current empirical literature with specific attention to the theoretical frameworks that have been used to illustrate how and why student learning occurs in interprofessional practice-based environments. Our findings show there are relatively few theoretical-based studies available to guide educators and researchers alike. We recommend a more considered and consistent use of theory and suggest that professional identity and socio-cultural frameworks offer promising avenues for advancing understandings of student learning and professional identity development within interprofessional practice-based environments.

## Background

There has been a proliferation of literature in the last ten years on interprofessional education and training models in clinical environments for pre-qualification and pre-registration health and social care students [1–13]. Although the primary focus of this has been on hospital ward settings where ‘students work in interprofessional teams, while under supervision, to manage the care of patients’ [4], this training model has also been used in community health care settings, such as primary care [14] and rehabilitation [15]. Thus practice-based (as opposed to ward-based) is a more inclusive term to use when referring to experiential models of interprofessional education and training involving the immersion of health and social care students in work-based settings. The research literature on practice-based interprofessional education and training has been mostly outcomes-oriented (mapped against Kirkpatrick’s framework of evaluation) and focused on evidencing the generally positive impact of such initiatives on student satisfaction, knowledge, skills, attitudes, and performance [16]. However, this literature does not evidence how and why learning occurs within interprofessional settings, and we have previously advocated using a realist approach to evaluation within the interprofessional education and training field [17] to address this shortcoming.

In this commentary we consider the empirical literature on student learning within interprofessional practice-based environments with two questions in mind, specifically:

1. What theoretical lenses have been used to help with understanding how and why student learning occurs within interprofessional practice-based environments?
2. What does the theoretical literature on interprofessional learning within practice-based environments imply for future directions in research?

In relation to question 1, although it is well established that we need to draw on theory to more fully understand the nature of interprofessional education, practice and care [18], there are only a handful of theoretical-based explanations reported in the literature about student learning within interprofessional practice-based environments. These include the recently published study by Conte et al*.* [19], which used socio-cultural learning theory as a lens to explore team collaboration during an interprofessional education rotation in intensive care. This study showed the importance of situated learning and guided participation within the clinical setting, and in particular the links between learner ownership and legitimate peripheral participation. By applying this particular theoretical lens, the authors were able to highlight the role played by supervisors in scaffolding or supporting student ‘ownership’ of learning and hence legitimate and peripheral participation in the clinical setting by knowing how and when to intervene. An alternative theoretical perspective is provided by Falk et al. [13], who used practice theory to analyse students’ collaboration and learning experiences in an interprofessional training ward. Their findings illustrated how students encountered particular tensions between expected and unexpected professional roles and responsibilities, leading the authors to suggest that practice-based interprofessional education and training settings can function as boundary zones. Using practice theory enabled the authors to illustrate how there can be conflicting and competing student expectations, motivations, and engagement within interprofessional training environments. Their study also showed the need to manage and leverage these expectations appropriately and scaffold practice in order to support meaningful and collaborative learning for all students involved [13]. Lastly, Hood et al. [11], observed that ‘learning and working together in an authentic clinical context allowed students to consider and to test the ‘ought’ self (who they think they are expected to be); the possible self (who they might be); and the desired self (who they would like to be)’, specifically their future professional identity. Despite this promising avenue of inquiry regarding professional identity, the authors did not fully expand on their observations or discuss how identity as a theoretical lens could frame understandings of student learning within interprofessional practice-based settings.

Given this lack of theoretically based explanations, we need to extend our gaze more broadly to other areas of the literature for theoretical guidance and for future research directions. One promising theoretical lens as alluded to by Hood et al*.* [11] is professional identity. This concept has begun to occupy an increasingly prominent position within the health professions education literature, and reflects a growing recognition that a fundamental goal of educating and training health professionals should be to facilitate and support the development of their professional identity [20, 21]. Professional identity not only draws attention to the process of ‘becoming’ a health professional, which involves both personal and professional identity dimensions [22]. It also prompts a focus on the interplay between identities and the possible dissonance among these [23] as students learn together within interprofessional practice-based environments characterized by teamwork and collaborative practice.

Furthermore, resonating with Conte et al. [19] we also suggest that socio-cultural frameworks can provide useful insights not just into the situated and relational nature of learning, within real-world interprofessional settings, but also into how students may construct and negotiate their professional identity. Understanding how students traverse professional boundaries and identities has emerged from preliminary work exploring health professional education in longitudinal integrated clinical placements where up to 30 students from different professions were placed at any one time in a rural community [24]. Although focussing on the perspective of the medical students, this study showed that it was the informal curriculum [25] comprising of multiple encounters between students, patients and their families, clinical teachers and other health staff, which played an important role in developing the students’ sense of preparedness for practice and professional identity as a rural practitioner. This study drew on Wenger’s notion that learning can be conceptualised as a social phenomenon reflecting ‘our own deeply social nature as human beings capable of knowing’, and as occurring within a social learning system (SLS) involving communities of practice, boundary processes, and identity formation [26]. A community of practice (CoP) [26] is the basic unit of analysis within a social learning system, and is defined as a ‘group of people who share a concern or a passion for something they do and learn how to do it better as they interact regularly’. These CoPs can be understood as the learning spaces in which competence and identity are developed, and individual learners participate in multiple CoPs and negotiate the boundaries between them in different ways [27].

We do acknowledge that many of the clinical environments in which students find themselves are unlikely to have been designed around the principles of interprofessional collaborative practice. This doesn’t mean that students are exposed to a null curriculum, where interprofessionalism is deliberately avoided in learning and teaching, and students are given the message that it is not important. [28] Rather, the richness of the informal curriculum in longitudinal placements, where students may also socialise within the local community that hosts their learning, appears to provide a learning space where interprofessional learning is inevitable. [29]. Emerging evidence of the utility of longitudinal integrated clinical placements in facilitating, for example, the acquisition of teamwork competencies [30] suggests that such placements are one way of promoting the development of interprofessionalism, where two or more professions are co-located.

## Conclusion

In this commentary we have raised some preliminary questions about what theoretical lens have (and can be) usefully applied to understand how and why student learning occurs within interprofessional practice-based environments. We hope this discussion is helpful in prompting educators and researchers alike to consider the theoretical underpinnings of educational design and research in the field. What remains unequivocally clear is that theoretically informed explanatory models are urgently required to continue to advance understandings of the nature of students’ learning and professional identity development within interprofessional practice-based environments.
